# Different Clinical Outcomes of COVID-19 in Two Healthcare Workers Vaccinated with BNT162b2 Vaccine, Infected with the Same Viral Variant but with Different Predisposing Conditions for the Progression of the Disease †

**DOI:** 10.3390/vaccines10020298

**Published:** 2022-02-15

**Authors:** Loredana Alessio, Mariantonietta Pisaturo, Antonio Russo, Lorenzo Onorato, Mario Starace, Luigi Atripaldi, Nicola Coppola

**Affiliations:** 1 Department of Mental Health and Public Medicine, Section of Infectious Diseases, University of Campania L. Vanvitelli, 80138 Naples, Italy; loredana.alessio@policliniconapoli.it (L.A.); mariantonietta.pisaturo@unicampania.it (M.P.); antonio.russo2@unicampania.it (A.R.); lorenzoonorato@libero.it (L.O.); mariostarace1984@libero.it (M.S.); 2 U.O.C. di Patologia Clinica Ospedale D. Cotugno, Azienda Sanitaria Ospedali dei Colli, 80131 Naples, Italy; luigi.atripaldi@ospedalideicolli.it

**Keywords:** COVID-19, SARS-CoV-2, BNT162b2, mRNA vaccine, B1.1.7, clinical outcome

## Abstract

Safe and effective vaccines are available to face the global threat of the COVID-19 pandemic. In this article, we report on the clinical cases of two healthcare workers vaccinated with two doses of BNT162b2 vaccine who were infected by the same viral clade but had different clinical outcomes.

## 1. Introduction

Since December 2019, the world has been facing a pandemic of unprecedented proportions due to a novel betacoronavirus, the severe acute respiratory syndrome coronavirus-2 (SARS-CoV-2), which has caused almost 200 million cases of infection and over 4 million deaths up to July 2021 [1]. Luckily, the availability of safe and effective vaccines is going to change the global epidemiology of the infection [2]. For both mRNA vaccines approved, efficacy of almost 95% was demonstrated for the prevention of symptomatic COVID-19 cases in two Phase 3 trials [3,4]. Moreover, a recently published retrospective cohort study with more than 20,000 patients demonstrated that receiving at least one dose of either vaccine was associated with a 100% reduction in the rate of hospitalization or death, including in a fragile population of patients with liver cirrhosis [5]. Finally, a case–control study reported that a two-dose vaccination with BNT162b2 retained a high effectiveness in the prevention of symptomatic cases, including against the feared B.1.617.2 (Delta) variant [6].

In the present paper, we report the virological and clinical characteristics of two healthcare workers who had both received two doses of BNT162b2 vaccine and were infected with the same viral strain but with different clinical progression. 

## 2. Clinical Cases

Case 1 was a 59-years-old male who suffered from lymphoproliferative disease of granular lymphocytes (LDGL). For this disease, he had undergone a bone marrow biopsy in February 2021 and started treatment with prednisone on 6 April 2021. He received the first dose of COVID-19 mRNA vaccine BNT162b2 on 12 January 2021 and the second dose on 2 February 2021. Case 2 was a 59-year-old female with a history of arterial hypertension, compensated due to therapy. She received the first and second dose of COVID-19 mRNA vaccine BNT162b2 on the same days as Case 1. Cases 1 and 2 shared the same room at work. On 12 April 2021, Case 1 showed an antispike titer of 122 binding arbitrary units (BAU)/mL (normal value under 33.8; LIAISON SARS-CoV-2-trimeric S IgG, Diasorin, Saluggia, Italy); Case 2 showed an antispike titer of 899 BAU/mL.

**Figure 1:**
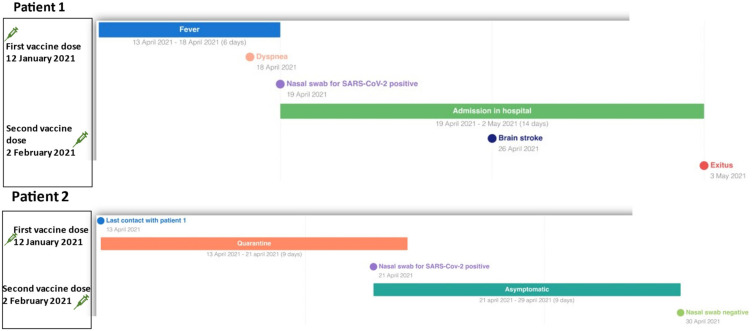
The clinical history of Cases 1 and 2.

Figure 1 shows the clinical history of Cases 1 and 2 (Figure 1).

On 14 April 2021, Case 1 showed the onset of fever (38–38.5 °C) and cough for which therapy was started with amoxicillin/clavulanate and continued with prednisolone. On 19 April 2021, due to the persistence of symptoms and the development of dyspnea, a nasopharyngeal swab was performed, which was positive for SARS-CoV-2-RNA. He was admitted to the COVID-19 emergency room. A high-resolution computerized tomography tested positive, with a score of 19/25 according to the Yang severity score [7]. On the same day, he was admitted at our COVID-19 unit in severe condition with 92% O_2_ saturation in nasal cannulas at 6 liters/minute (fraction of inspired oxygen (FiO_2_): 0.44%) (P/F 141), heart rate 75 beats per minute, and blood pressure 135/70 mmHg. He was therefore put on a high flow nasal cannula (HFNC), 60% FiO_2_, 60 L/min, obtaining peripheral saturation of 97%. The patient began treatment with methylprednisolone 40 milligrams twice per day, enoxaparin sodium 6000 international units (IU) per day, and pantoprazole 40 milligrams per day. On day 2 of hospitalization, despite the high flow nasal cannula, the patient presented desaturations and was therefore placed in a helmet with continuous positive airway pressure (CPAP), 55% FiO_2_, and positive end expiratory pressure (PEEP) 7.5 cm H_2_O, with a rapid improvement in clinical parameters (peripheral hemoglobin saturation 99%) and hemogasanalytic parameters pH 7.49, PCO_2_ 41.4, PO_2_ 227, and P/F 412. The following days, diurnal CPAP with helmet and HFNC night cycles were applied with a good clinical response. 

On day 6 of hospitalization, he reported headache and hemianopsia in the left eye, for which a CT brain scan showed an ischemic lesion in the territory of the right posterior cerebral artery. As he was not a candidate for thrombolysis, acetylsalicylic acid was added to the therapy. During the same day, the patient reported a subjective improvement in neurological symptoms and continued ventilatory and oxygen therapy alternating helmet CPAP and HFNC. After 2 days, a control CT showed stability of the lesions described above. On 29 April 2021 at 6:00 a.m. the patient had dysarthria, hemiparesis, and a worsening of peripheral saturation and blood gas analysis parameters important to reach an increase of FiO_2_ to 78%. The brain CT showed an increase in the acute ischemic lesion in the left temporoparietal region, subcortical area, extending to the ipsilateral deep white substance and responsible for compression of the right ventricle. Due to the neurological and respiratory conditions, the patient was transferred to an intensive care unit, where he died on 3 May 2021.

Case 2 had close contact with Case 1 up to 15 April 2021. Since the evidence of positivity for SARS-CoV-2 of Case 1 and in the absence of signs and symptoms, a nasopharyngeal swab for SARS-CoV-2-RNA was performed and showed a positive result. She remained at all times symptom-free at in home isolation up to 1 May 2021, when she became negative for nasopharyngeal swab for SARS-CoV-2.

The analysis of the SARS-CoV-2 RNA isolated from the nasopharyngeal swabs of both cases showed the presence of the same viral clade, the VUI202012/01 GRY (B.1.1.7) viral variant, named the Alpha variant. In Case 1, the threshold cycle (CT) of the RT-PCR was 21 for ORF 1ab, N and E genes. In Case 2, the CT was 28 for ORF 1ab, N and E genes. Furthermore, the neighbor joining and maximum likelihood methods were performed to evaluate the similarity and evolution analysis (Supplementary Figure S1). 

## 3. Molecular Methods

The RNA was pulled out by nasopharyngeal swabs of the two patients using QIAamp Viral RNA Mini Kit (Qiagen, Hilden, Germany) according to the manufacturer’s protocol. The concentration and quality of all extracted RNA samples were measured and checked with Nanodrop2000 (Thermo Fisher Scientific, Waltham, MA, USA). 

Viral genomes were amplified by performing a multiplex approach using version 1 of the CleanPlex SARS-CoV-2 Research and Surveillance Panel (Paragon Genomics, Hayward, CA, USA) following the manufacturer’s protocol, starting with 50 ng of total RNA and followed by Illumina sequencing on a NextSeq 500 (Illumina, San Diego, CA, USA). 

Libraries were controlled with High-Sensitivity Labchip and quantified with the Qubit Fluorometric Quantitation system (Thermo Fisher Scientific, Waltham, MA, USA). 

Raw data were trimmed and analyzed by the popular CLC workbench 5 bioinformatics software and Basic Local Alignment Search Tool (BLAST). Italian sequences imported into GeneBank database (https://www.ncbi.nlm.nih.gov/genbank; last accessed on 31 May 2021) from March 2020 to May 2021 and with the released accession numbers were used to draw phylogenetic trees. In addition, MEGA X software was used to obtain multiple sequence alignment (MSA), and the phylogenetic trees were drawn using the 1000 replicate bootstrap method.

## 4. Discussion

SARS-CoV-2 is a betacoronavirus transmitted from human to human through nasal or oral droplets or through close contacts [8,9]. After a mean incubation period of 5.2 (range 2–14) days, SARS-CoV-2 infection ranges from asymptomatic/mild forms in nearly 80% of infected subjects to moderate forms in about 15% and may lead to severe clinical presentation in the remaining 5% of subjects [10]. 

From clinical observation, several risk factors for poor prognosis and high mortality have been identified, such as age, hypertension, obesity, diabetes, and cancer [11,12,13]. Older adults are particularly susceptible to COVID-19 disease due to the presence of multiple comorbidities and chronic diseases [12] and more frequently present serious complications for which hospitalization in intensive care unit (ICU) is required [11,12,13].

This report describes different COVID-19 outcomes in two Italian healthcare workers, both immunized with the same type of vaccine on the same days and both infected by the same virus as established by the virological analysis. Case 1, with a chronic hematological disorder at the time of immunization, had a fatal outcome of COVID-19. Case 2, without a hematological or immunological disorder, remained paucisymptomatic, although both harbored the same viral clade. 

The vaccination program in Italy started in late December 2020, with priority given to healthcare workers, nursing home residents, and people over 80 years of age; the Pfiz-er/BioNTech BNT162b2 mRNA vaccine was used [14].

A prospective study enrolling a cohort of healthcare workers undergoing regular asymptomatic testing in England documented 70% effectiveness (95% CI 55–85) of a single dose of BNT162b2 vaccine in reducing the infection rate at 21 days, with an 85% incidence reduction of cases reported at 7 days after the second dose [15]. Moreover, the proportion of patients with typical COVID-19 symptoms was 66.6% in the 816 unvaccinated patients with available data compared to 36% among the vaccinated cohort [15].

There are concerns about efficacy of the vaccines against emergent SARS-CoV-2 viral variants, such as B.1.1.7 (N501Y. V1), known as the Alpha variant, harbored in the cases described in the present paper. Collier et al. analyzed neutralization of a pseudovirus including the spike protein with the full set of mutations present in the B.1.1.7 viral variant by sera collected after the first and second doses of the BNT162b2 vaccine and by convalescent sera. A small reduction using sera from vaccinated individuals, especially after the first dose, was observed; this reduction was also evident using convalescent sera [16].

Few data are available in the literature regarding the effectiveness of anti-SARS-CoV-2 vaccine in patients with immunodepression. In this regard, it is interesting to note the results of a real-world study performed in Israel showing a lower efficacy of BNT162b2 mRNA against infection in immunocompromised patients (71%, 95% CI: 37–87% and 52%, 95% CI: −26–82% in those who were 65 and older), while the overall efficacy was 90% (95% CI: 79–95%) in nonimmunocompromised patients [17]. Instead, the efficacy of BNT162b2 mRNA seems to be similar in the HIV population and in healthcare workers. In fact, neutralizing antibodies were detected in 131 (97%) of 135 HIV subjects and in 197 (98%) of the 201 healthcare workers [18]. 

Our Case 1 had a hematological disorder that made him more fragile against the disease than his colleague infected with the same virus. In fact, he showed a state of hypercoagulability that triggered ischemic cerebral events that, together with bilateral interstitial pneumonia, led to the patient’s death. Thus, in this patient, the approach used to ensure immunization failed. It is hypothesized that this was caused by the hematological disorder which the subject chronically suffered from and which probably reduced the mounting of the immune response induced by the vaccine towards the virus despite an antispike titer of 122 BAU/mL. In fact, it is inconceivable that the severity of COVID-19 was caused by a greater virulence of the viral strain or by a viral escape at vaccination because the colleague infected with the same viral strain and immunized with the same vaccine in the same period had a paucisymptomatic form of COVID-19. 

Many studies have shown a more severe clinical form of COVID-19 in patients with cancer compared to those without [19,20,21,22]. A recent meta-analysis including 32 studies on 46,499 patients, of whom 1776 had cancer, described a higher rate of mortality for all cause (RR 1.66; 95% CI 1.33–2.07, *p* < 0.0001) and a greater need for admission in ICU (RR 1.56; 9%% CI 1.31–1.87, *p* < 0.0001) in oncologic versus nononcologic patients. A multicenter cohort study on 928 patients with active or previous cancer and with confirmed SARS-CoV-2 infection showed a 30-day mortality for all cause for 13% of this population and, among the factors independently associated with mortality, accounted for the presence of an active cancer (OR 5.20, 995% CI 2.77–9.77) [22]. Finally, in a cohort of 371 COVID-19 patients, Monari et al. [13] compared 12 patients with nonactive cancer to 337 without cancer. The 17 patients with active cancer showed a higher rate of severe COVID-19 and mortality.

In this setting, there have been reports showing the response to vaccines in subjects with hematological cancer [23,24,25,26,27,28], such as Case 1 in this study. For example, Benda et al. observed a total of 259 hemato-oncological patients vaccinated with two 30 µg doses of BNT162b2 administered 21 days apart. Seven weeks after the first dose, spike protein receptor binding domain (S/RBD) antibodies were detected in 71.4% of the 123 hematological and in 94.5% of the 136 oncological patients (*p* < 0.001). Moreover, the hematological patients receiving systemic treatment had a 14.2-fold increased risk of not responding (95% confidence interval: 3.2–63.3, *p* = 0.001) [23]. Pimpinelli et al. described the safety and immunogenicity of BNT162b2 mRNA vaccine in hematological patients. In particular, the authors evaluated 42 patients with multiple myeloma (MM) and 50 patients with myeloproliferative malignancies (MPM), all of them on active anticancer treatment, and compared them with 36 elderly controls without cancer. Results showed the seroprotection rate was 100% in controls compared to 78.6% in MM (*p* = 0.003) and 88% in MPM (*p* = 0.038) patients [24].

The description of these clinical cases is a stimulus to continue the vaccination campaign set up for individuals not affected by immunological disorders but opens the way for new scenarios to identify new immunization strategies in subjects with hematological disorders and immune deficiencies.

## Supplementary Materials

The following supporting information can be downloaded at: https://www.mdpi.com/article/10.3390/vaccines10020298/s1, Figure S1: Phylogenetic tree for 39 complete genome of the SARS-CoV-2 using neighbor joining method and 1000 bootstrap, the black triangle indicates two cases analysed.
