# Supplementary material for: Different Clinical Outcomes of COVID-19 in Two Healthcare Workers Vaccinated with BNT162b2 Vaccine, Infected with the Same Viral Variant but with Different Predisposing Conditions for the Progression of the Disease
Source: Vaccines (Basel). 2022 Feb 15;10(2):298. doi: 10.3390/vaccines10020298 (PMC8877805; doi:10.3390/vaccines10020298)

DIFFERENT CLINICAL OUTCOME OF COVID-19 IN TWO HEALTHCARE WORKERS VACCINATED WITH BNT162B2 PFIZER/BIONTECH VACCINE AND INFECTED WITH THE SAME VIRAL VARIANT, B.1.1.7 (ALPHA)

SUPPLEMENTARY DATA

Molecural methods

The RNA was extracted from the nasopharyngeal swabs of the two patients using QIAamp Viral RNA Mini Kit (Qiagen, Hilden, Germany) following manufacturer's protocol. Both the concentration and the quality of all isolated RNA samples were measured and checked with the Nanodrop2000 (Thermo Fisher Scientific, Waltham, United States)

Viral genomes were amplified by using a multiplex approach, using version 1 of the CleanPlex SARS-CoV-2 Research and Surveillance Panel (Paragon Genomics, Hayward, United States), according to the manufacturer's protocol starting with 50ng of total RNA and followed by Illumina sequencing on a NextSeq 500 (Illumina, san Diego, United States).

Libraries were checked using High Sensitivity Labchip and quantified with Qubit Fluorometric Quantitation system (Thermo Fisher Scientific, Waltham, United States). Raw data were trimmed and analyzed using popular bioinformatics software CLC workbench 5, and Basic Local Alignment Search Tool (BLAST). Italian sequences submitted into GeneBank database (https://www.ncbi. nlm.nih.gov/genbank) from March 2020 to May 2021 and released accession numbers were used to draw phylogenetic trees. Moreover, MEGA X software was used for multiple sequence alignment (MSA) and the phylogenetic trees were drawn using the 1000 replicate bootstrap method.

Figure legend

Supplementary Figure S1: Phylogenetic tree for 39 complete genome of the SARS-CoV-2 using neighbor joining method and 1000 bootstrap, the black triangle indicates two cases analysed

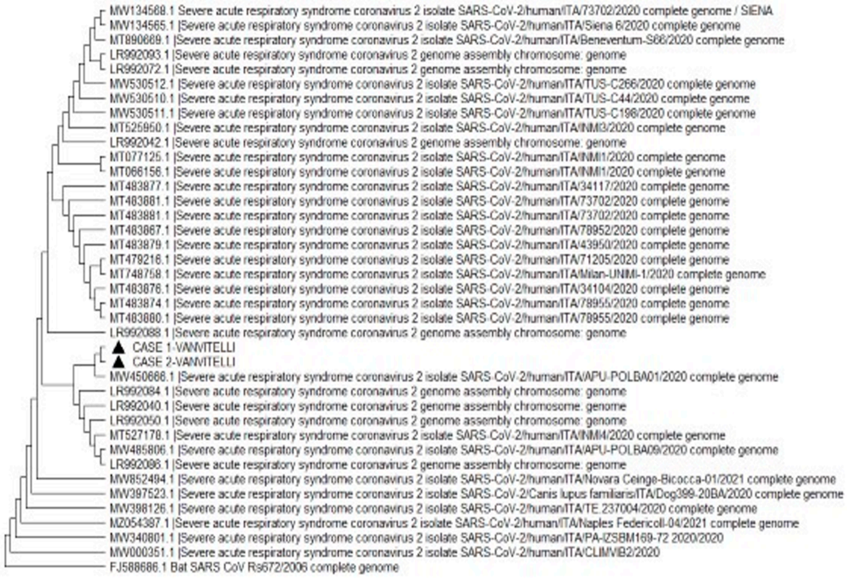

Supplement: Supplementary file 1 [file vaccines-10-00298-s001.zip › vaccines-1562275-supplementary.pdf]
